# Laboratory transmission potential of British mosquitoes for equine arboviruses

**DOI:** 10.1186/s13071-020-04285-x

**Published:** 2020-08-12

**Authors:** Gail E. Chapman, Ken Sherlock, Jenny C. Hesson, Marcus S. C. Blagrove, Gareth J. Lycett, Debra Archer, Tom Solomon, Matthew Baylis

**Affiliations:** 1 Epidemiology and Population Health, Institute of Infection and Global Health, University of Liverpool, Liverpool, UK; 2 Vector Biology, Liverpool School of Tropical Medicine, Liverpool, UK; 3 Institute of Infection and Global Health, University of Liverpool, Liverpool, UK; 4 Health Protection Research Unit in Emerging and Zoonotic Infections, University of Liverpool, Liverpool, UK; 5 Walton Centre NHS Foundation Trust, Liverpool, UK

**Keywords:** JEV, RRV, VEEV, Arbovirus, *Aedes*, *Ochlerotatus*, *Culex*, *Culiseta*, Mosquito, Vector competence

## Abstract

**Background:**

There has been no evidence of transmission of mosquito-borne arboviruses of equine or human health concern to date in the UK. However, in recent years there have been a number of outbreaks of viral diseases spread by vectors in Europe. These events, in conjunction with increasing rates of globalisation and climate change, have led to concern over the future risk of mosquito-borne viral disease outbreaks in northern Europe and have highlighted the importance of being prepared for potential disease outbreaks. Here we assess several UK mosquito species for their potential to transmit arboviruses important for both equine and human health, as measured by the presence of viral RNA in saliva at different time points after taking an infective blood meal.

**Results:**

The following wild-caught British mosquitoes were evaluated for their potential as vectors of zoonotic equine arboviruses: *Ochlerotatus detritus* for Venezuelan equine encephalitis virus (VEEV) and Ross River virus (RRV), and *Culiseta annulata* and *Culex pipiens* for Japanese encephalitis virus (JEV). Production of RNA in saliva was demonstrated at varying efficiencies for all mosquito-virus pairs. *Ochlerotatus detritus* was more permissive for production of RRV RNA in saliva than VEEV RNA. For RRV, 27.3% of mosquitoes expectorated viral RNA at 7 days post-infection when incubated at 21 °C and 50% at 24 °C. Strikingly, 72% of *Cx. pipiens* produced JEV RNA in saliva after 21 days at 18 °C. For some mosquito-virus pairs, infection and salivary RNA titres reduced over time, suggesting unstable infection dynamics.

**Conclusions:**

This study adds to the number of Palaearctic mosquito species that demonstrate expectoration of viral RNA, for arboviruses of importance to human and equine health. This work adds to evidence that native mosquito species should be investigated further for their potential to vector zoonotic mosquito-borne arboviral disease of equines in northern Europe. The evidence that *Cx. pipiens* is potentially an efficient laboratory vector of JEV at temperatures as low as 18 °C warrants further investigation, as this mosquito is abundant in cooler regions of Europe and is considered an important vector for West Nile Virus, which has a comparable transmission ecology.
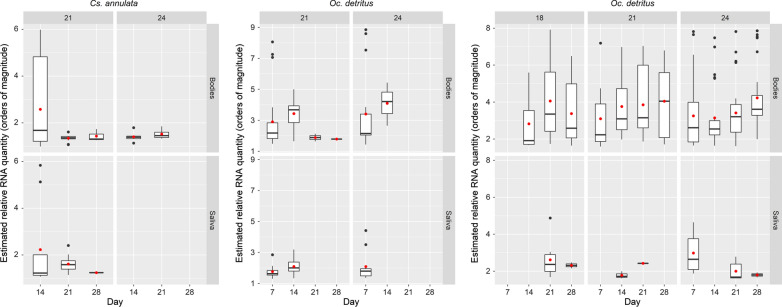

## Background

Globalisation and climate change are expected to change the level of risk for emergence of vector-borne diseases in previously unaffected regions. In the last fifty years, the geographical range of a number of mosquito-borne arboviral diseases has increased, including Zika, dengue, chikungunya and West Nile. Mosquito-borne arboviral infections which affect both horses and people include, amongst others, the flaviviruses West Nile virus (WNV), Japanese encephalitis virus (JEV) and Murray Valley encephalitis virus (MVEV), and the alphaviruses Venezuelan equine encephalitis virus (VEEV), Eastern equine encephalitis virus (EEEV), Western equine encephalitis virus, and Ross River virus (RRV) [1]. Whilst the emergence in Europe of dengue and chikungunya has been associated with *Aedes aegypti* and the invasive mosquito *Aedes albopictus* [2], for most of the equine viruses *Culex* mosquitoes are significantly involved in transmission. Expansion of the range of some arboviruses (West Nile virus (WNV) for example) has demonstrated vector competence of previously naïve mosquito species or populations [3–6]. Other emerging diseases that affect equines include Peruvian horse sickness virus [7] and Bunyamwera virus [8, 9]. Both are mosquito-borne viruses that have emerged as fatal equine diseases, in Peru and Argentina respectively, within the last 25 years. Sindbis and Middelburg viruses, circulating in Europe and/or Africa have also been recently associated with neurological disease in horses [10].

There has been much discussion of the risk of equine arbovirus introduction to Europe in the last decade [11–14]. The equine arboviruses generally have complex enzootic transmission cycles involving wildlife as reservoir hosts and ‘bridge vectors’ with broad feeding preferences which can carry virus from the reservoir host to other hosts; including humans and horses, both of which are clinically affected. The three viruses investigated in the present study (VEEV, RRV, JEV) have significant impacts on the health of people and horses (summarised in [15]) in endemic areas. Venezuelan equine encephalitis virus circulates in enzootic cycles between rodent hosts and mosquito vectors in Mexico, Central and South America [16] and has a complex transmission cycle involving regular mutation of the virus, facilitating transmission to humans and horses through broadening of the vector and host ranges. This results in an epizootic cycle during which, virus amplification in the horse is sufficient to result in mosquito infection and this is thought to significantly increase the risk of human infection [17]. VEEV infection causes neurological signs in humans and horses and significant infection and mortality rates in horses [15, 18].

Ross River virus is active seasonally in Australia with a number of vectors implicated. Epidemic polyarthritis due to RRV infection is regularly encountered in people in Australia [19], and related signs are seen in infected horses including synovial effusion, muscle stiffness and exercise intolerance [20]. In Australia, RRV is maintained in a transmission cycle between mosquito vectors and marsupial hosts. However, a large outbreak occurred in the South Pacific in 1979–1980 [21], and other outbreaks consistent with human-mosquito-human transmission [22] provide evidence that regions without native marsupial hosts may be at risk of limited epizootic outbreaks. The predominance of marsupials as reservoirs of RRV has been called into question and horses are suggested as potentially significant reservoirs by some authors [23]. These factors raise the possibility that the potential for RRV to spread globally may be greater than previously thought.

Japanese encephalitis virus outbreaks have occurred from Asia to Oceania [1] and the virus infects a broad range of species although the primary transmission cycle involves ardeid birds [24]. JEV infection causes neurological disease and mortality in equines and humans. JEV has several secondary vectors as well as the main vector *Cx.* *tritaeniorhynchus*, and has been identified in numerous species of wild-caught mosquitoes including *Cx.* *pipiens*, in which JEV RNA was discovered in Italy in 2011 [25–27]. *Culex pipiens* has also been shown to be a laboratory competent vector, as has the invasive mosquito *Ae. albopictus* [28] which is widespread in southern Europe [29].

None of these three viruses have been identified in the UK; however, to estimate the risk of autochthonous transmission (post-introduction) of these viruses in an unaffected country it is necessary to consider potential native vectors. Several studies have investigated vector competence of European mosquitoes for WNV [30, 31], including UK populations [32], and for JEV [28, 33, 34]. While some mosquito species present both in Europe and the Americas or Oceania have had their vector competence assessed for equine alphaviruses such as VEEV and RRV [35, 36], to our knowledge no field-collected European mosquito populations have been experimentally evaluated for alphaviruses affecting equines.

The aim of this study was to investigate British wild-caught mosquito species for laboratory transmission potential (detection of viral RNA in saliva) of selected equine arboviruses, at temperatures which occur in the UK now, or may in the future. Viruses (an epidemic strain of VEEV, RRV and JEV) were selected based on their effects on equine health [15]. Mosquito species were selected based on the potential exposure of British equines to the candidate vector. During a previous study, UK equine premises were sampled for candidate mosquito vectors and *Cx. pipiens*, *Culiseta annulata* and *Oc. detritus* were collected on a significant number of sites [15].

The mosquito-virus combinations tested were JEV in *Cs. annulata* and *Cx. pipiens*, and RRV and VEEV in *Oc. detritus*. *Ochlerotatus detritus* has previously been shown to be a potential laboratory vector of JEV [34] and so was not further tested. None of these mosquito-virus combinations have been tested before, except for JEV and *Cx. pipiens*, which was examined here at a significantly lower temperature than previously [28].

The presence of viral RNA in saliva is a pre-requisite for a species being a vector, although this alone does not prove that a species is able to transmit under natural conditions. Hence, where viral RNA is detected in saliva, we refer to this as (laboratory) transmission potential to differentiate our results from laboratory vector competence demonstrated by transmission to vertebrates, and from natural transmission. Additionally, we use the term candidate vector to describe mosquito species with ecological characteristics such as host-preference and habitat type which make them of interest for vector competence evaluation.

## Methods

### Mosquitoes

Experiments were conducted on adult female mosquitoes originating from egg rafts or larvae collected on the Wirral Peninsula, northwest England. *Ochlerotatus detritus* were collected as third- or fourth-instar larvae, or pupae from brackish marshland by Little Neston (53°16′37.2″N, 3°04′06.4″W) between May and October. *Culex pipiens* egg-rafts were collected from container habitats on farmland at University of Liverpool, Leahurst Campus, Neston (53°17′25.6″N, 3°01′29.9″W), between May and August. *Culiseta annulata* egg-rafts were collected from container habitats (black 15 litre buckets were placed to catch rainwater and organic debris, for the purpose of attracting ovipositing *Cs. annulata*) in woodland at Ness Botanic Gardens, Little Neston (53°16′11.5″N, 3°02’48.3″W) between May and August. Individual egg-rafts were allowed to hatch in covered larval trays. *Culiseta annulata* egg rafts were initially differentiated from *Cx. pipiens* complex rafts based on size, and emerged adults were identified morphologically. To separate *Cx. pipiens* from the morphologically identical species *Cx. torrentium*, a small number of larvae hatched from each egg raft were identified to species level using restriction fragment length polymorphism analysis [37] and larval trays containing larvae identified as *Cx. pipiens* were retained. Immature mosquitoes were reared in a brick-built, unheated, non-insulated outbuilding (during May to November), thereby approximating outdoor shaded conditions. Larvae were reared in water collected from their larval habitat, supplemented with tap water as necessary. Where supplementary food was required Brewer’s Yeast was provided. Adults were allowed to emerge and mate in 30 × 30 × 30 cm BugDorms (BugDorm, Taichung, Taiwan). Adults were kept in ambient conditions (as for larvae) and were offered 10% sucrose solution on cotton wool *ad libitum*, then transferred to an indoor (temperature controlled) insectary on the same day as the virus-containing blood meal was offered.

### Viruses

Viruses used were the JEV strain CNS138-11 [38], RRV (National Collection of Pathogenic Viruses (NCPV) catalogue number 0005281v) and VEEV P676 (NCPV catalogue number 0605153v). All viruses were cultured and titre assayed in Vero cells. Final virus titre in blood meals was 1 × 10^6^ plaque forming units (pfu)/ml for JEV, 5.6 × 10^6^ 50% tissue culture infectious dose (TCID_50_)/ml for RRV, and 9.5 × 10^6^ pfu/ml for VEEV. Titres were chosen based on information about viraemia in amplification or transport hosts and previously published studies investigating laboratory transmission. Titres were limited by the stock concentration provided by the respective institutions (measured using plaque assay (JEV, VEEV) or endpoint dilution assay TCID_50_ (RRV)). Virus stocks were aliquoted on the day of receipt and stored at -80 °C, with aliquots discarded after use to minimise freeze-thaw before infection experiments.

### Infection

At 10–21 days post-emergence female mosquitoes were transferred into 1-litre polypropylene Dispo-safe containers (The Microbiological Supply Company, Luton, UK), with a fine mesh covering and were starved of sugar for 24 h. They were then allowed to feed for up to 3 h, in low light conditions at 21 °C, on heparinised human blood (NHS transfusion service, Speke, UK) containing the virus. A Hemotek membrane feeding apparatus (Discovery Workshops, Lancashire, UK) heated to 39 °C was used with the membrane provided by the manufacturer. Immediately before use this was worn next to human skin for 15–20 min, to impart human odour, and encourage feeding. Blood-fed females were incubated at 18 °C, 21 °C or 24 °C. Mosquitoes were maintained at this temperature for 7–35 days and were provided with 10% sucrose. On the day of testing, mosquitoes were immobilised with triethylamine (FlyNap, Carolina Biological Supply Company, Burlington, USA), and their saliva was extracted by inserting each mosquito’s proboscis into a capillary tube containing mineral oil for 30 min. Each mosquito and its expectorate were placed in a separate 1.5 ml microcentrifuge tube containing 200 µl TRIzol reagent (Thermo Fisher Scientific, Waltham, USA), kept at room temperature for 2 h to inactivate virus and then stored at − 20 °C. Repeat infections were carried out for each experimental condition if 30 surviving mosquitoes were not available for testing at all time points. In this case another batch of mosquitoes was infected, until no further mosquitoes of under 22 days post-emergence were available. Our intention was to analyse at least 30 surviving mosquitoes for each condition. Total numbers infected were not recorded due to accidental mortality.

### Measuring viral RNA in body and saliva

Semi-quantitative qPCR was used to estimate viral RNA quantities in mosquito saliva and bodies. Samples were run in duplicate and the mean of these two C_q_ values was used in further analysis (see Additional file 1: Table S1). RNA was extracted using TRIzol reagent as per the manufacturer’s instructions. Samples were stored at − 20 °C for up to 14 days before cDNA generation. cDNA was generated using Superscript™ Vilo™ (Thermo Fisher Scientific). Each 20 µl reaction consisted of 4 µl Superscript™ Vilo™ MasterMix, 6 µl RNase-free water, and 10 µl of sample. PCR plates were incubated at 25 °C for 10 min, then 42 °C for 90 min and the reaction was terminated at 85 °C for 5 min. cDNA was stored at − 20 °C.

TaqMan (Thermo Fisher Scientific) quantitative polymerase chain reaction (qPCR) was used to detect the presence of viral RNA in the samples. Primer and probe sets are shown in Table 1.

**Table 1:** Primer and probe sets for the TaqMan assays

Virus	Sense primer (5’-3’)	Probe (5’-3’)	Antisense primer (5’-3’)	Reference
VEEV	TCCATGCTAATGCYAGAGCGTTTTCGCA	Fam-TGATCGARACGGAGGTRGAMCCATCC-Tamra	TGGCGCACTTCCAATGTCHAGGAT	Vina-Rodriqez et al. [45]
RRV	TTGCCGGTGGGTAGAGAGAA	Fam-ACCACACTTTGGCGTAGAGC-Tamra	TCTGGCGGTGTATGCATGTC	This study^a^
JEV	ATCTGGTGYGGYAGTCTCA	Fam-CGGAACGCGAWCCAGGGCAA-Tamra	CGCGTAGATGTTCTCAGCCC	Pyke et al. [81]

^a^Designed using Primer-BLAST [82]

TaqMan qPCR assays were performed in a reaction volume of 20 µl. The reaction contained 1 × TaqMan Gene Expression Master Mix (with ROX passive reference), TaqMan probe (500 nM for VEEV and RRV assays; 150 nM for JEV assay), primers (1 µM for VEEV and RRV assays; 400 nM for JEV assay) and 2 µl of cDNA or control substance.

Thermocycler conditions for VEEV and RRV assays were: 1 cycle of 95 °C for 10 min, then 45 cycles of 95 °C for 15 s, 55 °C for 30 s and 60 °C for 30 s. For the JEV assay thermocycler conditions were: 1 cycle of 95 °C for 10 min, then 45 cycles of 95 °C for 15 s, and 60 °C for 1 min. Amplification and detection were performed using an Agilent Mx3005P qPCR System (Agilent Technologies, Santa Clara, USA).

### Analysis

For each cDNA generation, a no-template control (nuclease-free water), and a positive control (viral RNA) were assayed. For each TaqMan assay, a positive control (cDNA generated from neat virus RNA) and negative controls (nuclease-free water, and cDNA generated from a mosquito infected with JEV for VEEV and RRV assays or infected with VEEV for JEV assays) were included.

For each virus, a standard curve for the PCR was generated using 3 replicates of 10-fold serial dilutions with a dynamic range of 7 logs using the stock virus in order to allow calculation of estimated PCR efficiency (see Additional file 2: Text S1): JEV – 103.19%, RRV – 95.04%, VEEV – 91.66%.

The copy number of viral RNA in the stock virus was not known and therefore viral copy number cannot be estimated from C_q_ value. Samples were considered positive for viral RNA if the C_q_ value obtained from the sample was ≤ 40.

To aid the interpretation of C_q_ values on plots, an ‘estimated relative RNA quantity’ is represented for each viral RNA, on a scale showing orders of magnitude, relative to a sample producing a C_q_ value of 40. The method used here is semi-quantitative and the scales presented on plots correspond to transformed C_q_ values and not to absolute quantification of virus or RNA quantity (see Additional file 2: Text S1).

In this study, for percentage of mosquitoes with detectable viral RNA in bodies of saliva the denominator was the total number of mosquitoes successfully feeding on infected blood and surviving until the point of sampling.

All statistical analyses were performed using the statistical programming language R [39]. The difference in two proportions was analysed using Fisher’s exact test (fisher.test); the Shapiro-Wilks test was used to test whether data were normally distributed (shapiro.test). The Kruskal-Wallis rank sum test (kruskal.test) was used to test for significant differences in C_q_ values between groups, and pairwise Mann Whitney-U tests (wilcox.test) with a Holm correction [40], were used to test for significant differences between each pair of groups.

## Results

### Detection of JEV RNA in *Cs.* *annulata*

**Fig. 1:**
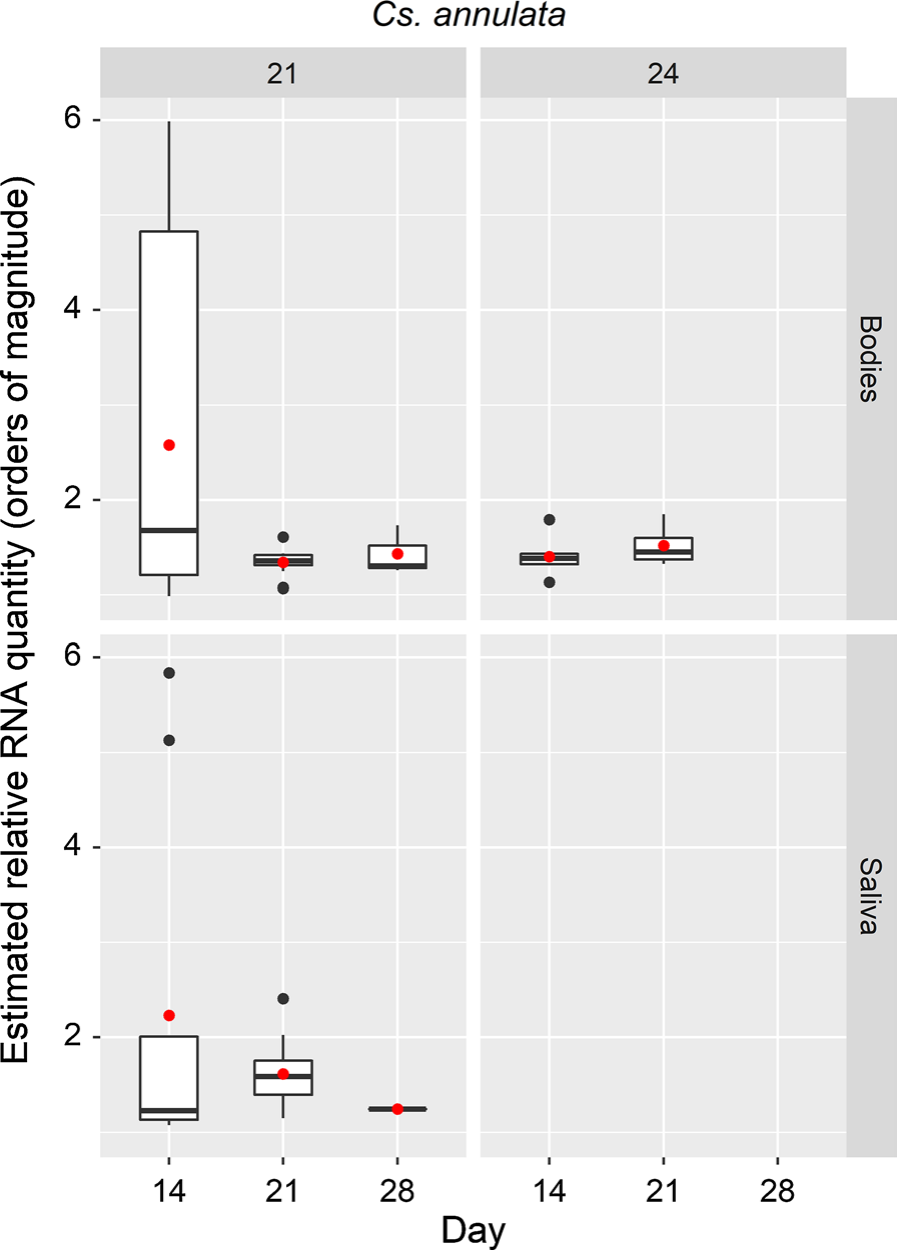
Box-and-whiskers plots for range of estimated relative quantities of JEV RNA in samples from *Cs.* *annulata*. Boxes indicate 2nd and 3rd quartiles, vertical lines upper and lower quartiles, and horizontal lines the median. Black points indicate outliers. Red points indicate mean values

*Culiseta* *annulata* was evaluated at 3 time points after challenge by ingestion with JEV and incubation at 21 °C and 24 °C (Table 2). The trend in percentage of mosquitoes with viral RNA in bodies and saliva was a reduction over time and both parameters are reduced at 24 °C compared to 21 °C. The range of estimated relative JEV RNA quantity for mosquito bodies and saliva is presented in Fig. 1.

**Table 2:** Summary of JEV RNA detection in saliva and bodies of *Cs. annulata*

Temperature (°C)	Timepoint (days)	Total no. of surviving mosquitoes	No. with detectable viral RNA in body	No. with detectable viral RNA in saliva	% with detectable viral RNA in body	% with detectable viral RNA in saliva
21	14	30	13	9	43.3	30.0
	21	35	20	15	57.1	20.0
	28	30	3	1	10.0	3.3
24	14	30	6	0	20.0	0
	21	24	4	0	16.7	0
	28	5	0	0	0	0

### Detection of JEV RNA in *Cx.* *pipiens*

A small number of *Cx.* *pipiens* were tested at one temperature (18 °C) and one time point (21 days). All 18 mosquitoes tested positive for viral RNA in bodies, and 13 (72.2%) had viral RNA in saliva. Median C_q_ values produced from mosquito bodies and saliva were 33.85 and 36.78 respectively.

### Detection of RRV RNA in *Oc.* *detritus*

*Ochlerotatus* *detritus* exposed to a blood meal containing RRV were evaluated at 5 time points after challenge, and incubation at 21 °C and 24 °C (Table 3). The percentage of mosquitoes expectorating viral RNA was highest after 7 days with an incubation temperature of 24 °C.

**Table 3:** Summary of RRV RNA detection in saliva and bodies of *Oc. detritus*

Temperature (°C)	Timepoint (days)	Total no. of surviving mosquitoes	No. with detectable viral RNA in body	No. with detectable viral RNA in saliva	% with detectable viral RNA in body	% with detectable viral RNA in saliva
21	7	33	23	9	69.7	27.3
	14	30	25	11	83.3	36.7
	21	30	2	0	6.7	0
	28	20	1	0	5.0	0
	35	10	0	0	0	0
24	7	22	16	11	72.7	50.0
	14	30	4	0	13.3	0
	21	30	0	0	0	0
	28	12	0	0	0	0
	35	15	0	0	0	0

**Fig. 2:**
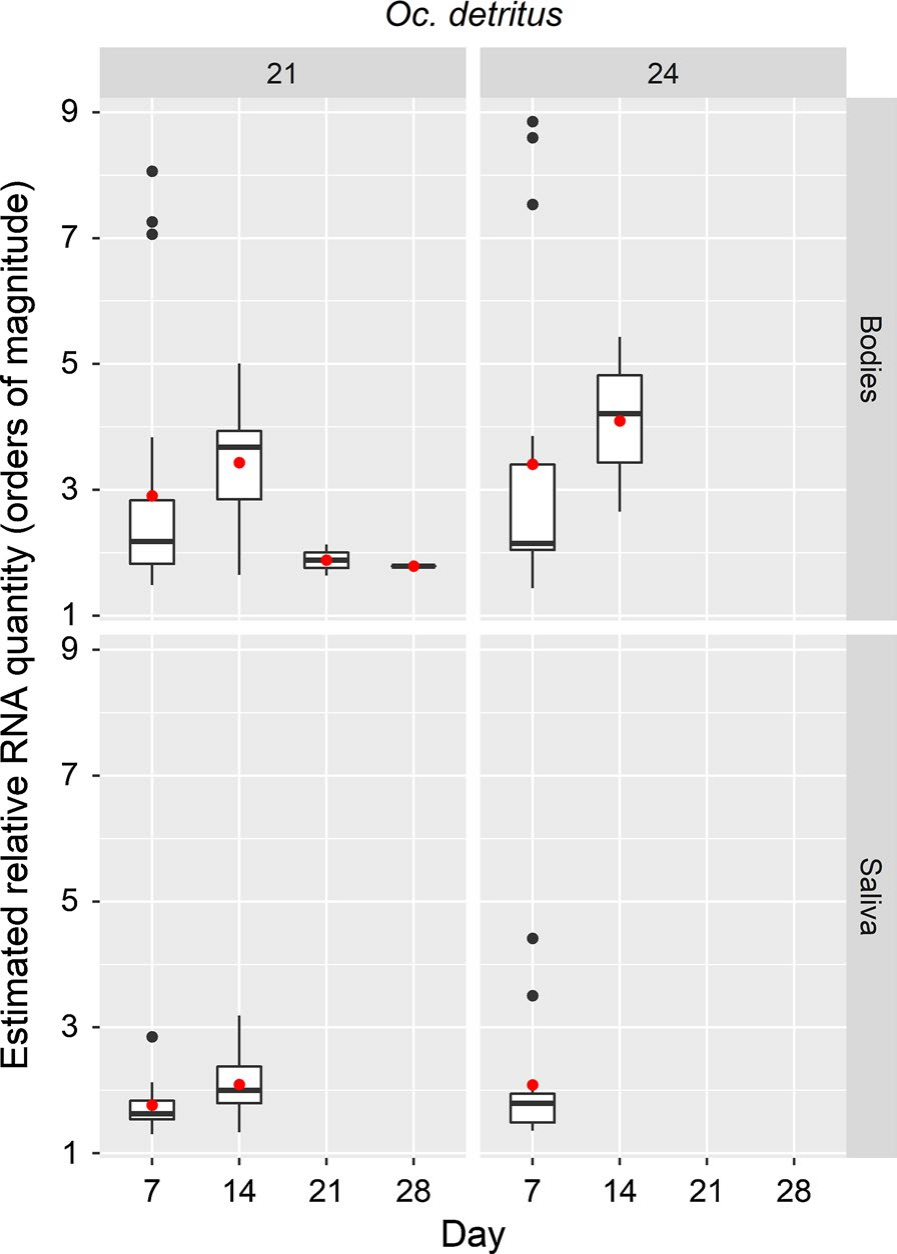
Box-and-whiskers plots for range of estimated relative RRV RNA quantity in samples from *Oc.* *detritus*. Boxes indicate 2nd and 3rd quartiles, vertical lines upper and lower quartiles, and horizontal lines the median. Black points indicate outliers. Red points indicate mean values

At both temperatures, by 21 days, the percentage of mosquitoes expectorating RRV RNA and the proportion with detectable viral RNA in their bodies had dropped significantly compared to those at 7 days (*P* < 0.001 in both cases). This observation correlates with the drop in estimated quantity of RNA detected in these bodies seen at 21 °C (Fig. 2).

C_q_ values were not normally distributed (Shapiro-Wilks test) and variances were significantly different between groups. There were significant differences between incubation periods in the C_q_ values of mosquito bodies maintained at 21 °C (*χ*^2^ =13.67, *df* = 3, *P* = 0.003). Pairwise tests indicated a significant difference in C_q_ values between mosquito bodies after a 14 day incubation period, compared to a 7 day incubation period at 21 °C (*P* = 0.012).

### Detection of VEEV RNA in *Oc.* *detritus*

**Fig. 3:**
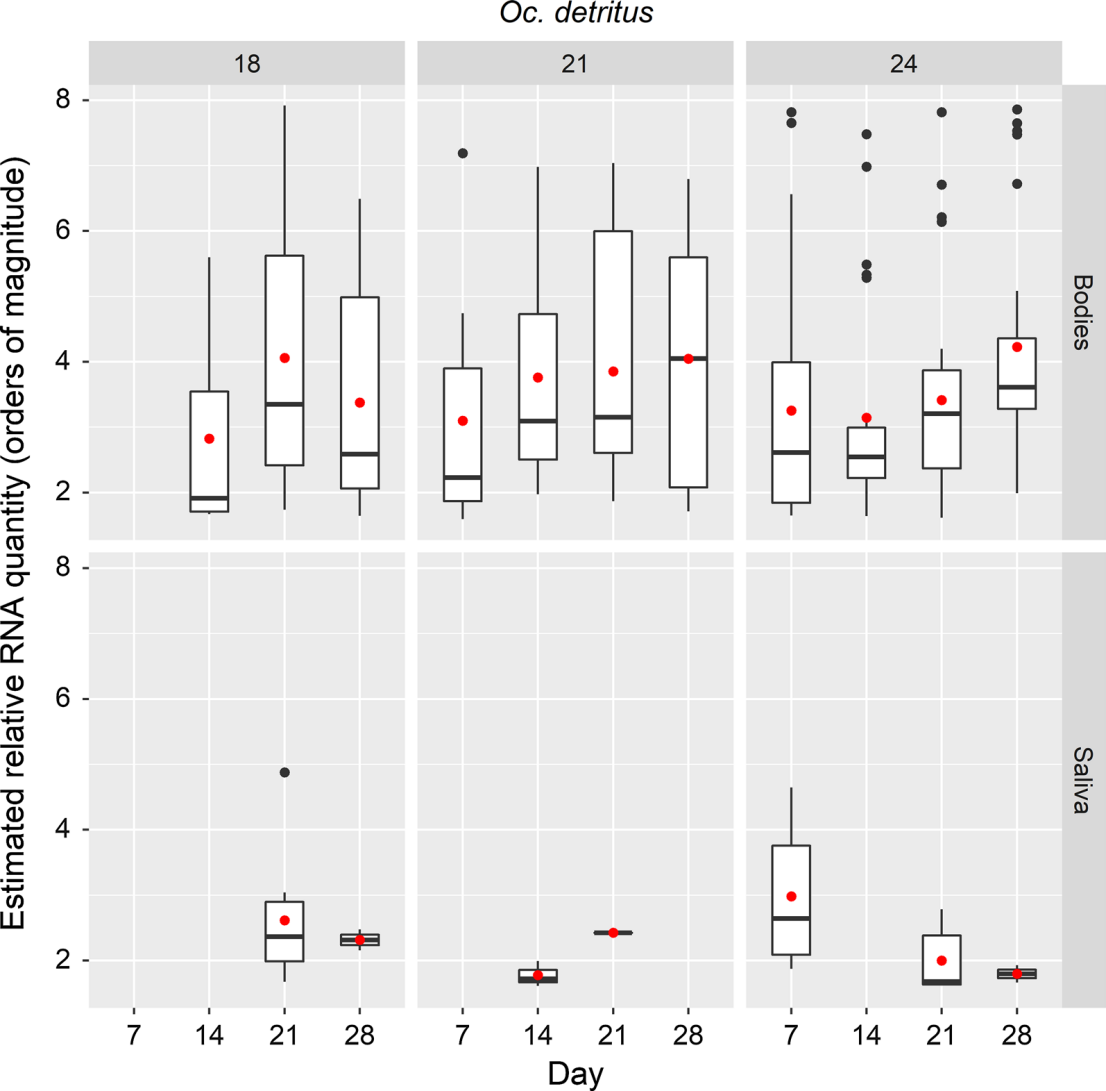
Box-and-whiskers plots for range of estimated relative values of VEEV RNA in samples from *Oc. detritus*. Boxes indicate 2nd and 3rd quartiles, vertical lines upper and lower quartiles, and horizontal lines the median. Black points indicate outliers. Red points indicate mean values

*Ochlerotatus.* *detritus* was evaluated at 4 time points after challenge by ingestion with VEEV and incubation at 18 °C, 21 °C and 24 °C (Table 4; only 3 time points for 18 C). In general, the trend over time is for increased proportions of mosquitoes with VEEV RNA detected in the body and higher estimated relative RNA quantities in bodies (Fig. 3), but few mosquitoes expectorated viral RNA.

**Table 4:** Summary of VEEV RNA detection in saliva and bodies of *Oc. detritus*

Temperature (°C)	Timepoint (days)	Total no. of surviving mosquitoes	No. with detectable viral RNA in body	No. with detectable viral RNA in saliva	% with detectable viral RNA in body	% with detectable viral RNA in saliva
18	14	25	3	0	12	0
	21	28	23	9	82.1	32.1
	28	30	23	2	76.7	6.7
21	7	29	11	0	37.9	0
	14	30	23	3	76.7	10.0
	21	28	25	1	89.3	3.6
	28	15	5	0	33.3	0
24	7	30	22	7	73.3	23.3
	14	28	25	0	89.3	0
	21	27	27	6	100.0	22.2
	28	30	27	1	90.0	3.3

## Discussion

Here we present the first demonstration of laboratory transmission potential of any European mosquito population for VEEV and RRV, with RNA of both viruses detected in *Oc.* *detritus* saliva. *Ochlerotatus detritus* has previously been shown to be able to produce flaviviruses in saliva [32]; to our knowledge, this is the first study demonstrating it can produce viral RNA of an alphavirus (RRV). Detection of VEEV RNA in the saliva of *Oc. detritus* was infrequent, despite high proportions of mosquito bodies being infected, marking this species as a potential but inefficient laboratory transmitter.

We found that over 70% of *Cx.* *pipiens* produced RNA of JEV in saliva after being maintained at 18 °C, similar to the mean temperature of some recent UK summer months. *Culex* *pipiens* has previously been shown to be capable of laboratory transmission of JEV after 11 days when maintained at 27 °C [28]. Lower incubation temperatures had not been previously used for this mosquito-virus pair. Further work in order to confirm laboratory competence and estimate the lower temperature limit for replication of JEV in *Cx. pipiens* is warranted as *Cx.* *pipiens* is a widespread vector of West Nile virus [41–43] and has been considered a potential vector of JEV in Europe [28].

We add *Cs. annulata* to the number of European species shown to produce JEV RNA in saliva. To our knowledge, *Cs.* *annulata* has only been tested once before for vector competence for an arbovirus [44]: it was shown to be competent but was not an efficient vector of Tahnya virus (Bunyaviridae; Orthobunyavirus).

The results of this study for RRV and *Oc. detritus,* and for JEV and *Cs. annulata,* differ from the familiar pattern of number of mosquitoes positive for viral RNA increasing over time and with temperature: (i) in some instances, a decrease in viral RNA titre at later time points; and (ii) a decrease in viral RNA titre with higher maintenance temperature. It seems plausible that for these non-naturally occurring mosquito-virus interactions we are able to generate infections that are unstable over time; with the virus killing the mosquito or the mosquito clearing the virus, both processes facilitated by higher temperatures or longer incubation. Unfortunately, survival rates in infected or non-infected mosquitoes was not investigated in this study. Thus, we are unable to ascertain whether viral infection was causing mosquito mortality, which could account for the detection of more uninfected mosquitoes at later time points.

An important question in mosquito infection studies is whether the titre of virus in the blood meal reflects the virus titre in natural hosts. If the experimental titres are much higher than occur naturally, demonstrating infectivity to the mosquitoes may not indicate true transmission potential. Estimated blood-meal virus titres of RRV (5.6 × 10^6^ TCID_50_/ml) and VEEV (9.5 × 10^6^ pfu/ml) used in this study were generally comparable to host viraemias. Reported titres of RRV include 1 × 10^5.5^ TCID_50_/ml in humans [45] and 1 × 10^6.3^ 50% suckling mouse intracerebral lethal dose (SMICLD_50_)/ml in horses [46]. Reported titres of VEEV in horses range from 1 × 10^5.3^ to 1 × 10^8.5^ SMICLD_50_/ml [47–49]. The estimated bloodmeal titre of JEV used here (1 × 10^6^ pfu/ml) exceeds that reported for natural hosts by one to two logs, such as pigs (1 × 10^4.5^ TCID_50_/ml [50] or 1 × 10^4^ SMICLD_50_/ml [51, 52]), ardeid birds (1 × 10^4.3^ SMICLD_50_ /ml [53]) and non-ardeid birds (1 × 10^5.4^ pfu/ml [54]). In the present study, titres in blood meals were estimated from frozen stock solution maximum titres. A previous study by our group using the same method of titre estimation for JEV with similar storage conditions, overestimated the titre in blood meals by 2 logs [55]; therefore it was considered likely that the final titre in blood meals used in this study would be, in reality, closer to 1 ×10^4^ pfu/ml than 1 × 10^6^ pfu/ml and therefore, would approximate the JEV titre in natural hosts.

Confirmation that a mosquito species is a laboratory competent vector ideally involves demonstration of transmission from one vertebrate host to another. The use of vertebrate hosts in vector competence experiments has diminished in recent years, due to animal welfare considerations, and therefore alternative methods have been developed as a proxy for natural transmission [56–59]. These include mosquito infection by artificial blood meal with a comparable viral titre to viraemias seen in vertebrate hosts, and transmission estimated by saliva extraction through forced salivation. Quantification of infectious virus in the expectorate of mosquitoes can be achieved using cell culture, however this is technically challenging [60] and was not possible in this study. The present study uses detection of viral RNA in saliva, thus the results should be interpreted with caution: while detection of viral RNA in saliva is an important finding [32, 60, 61], we have not yet demonstrated the production of infectious virus.

For demonstration of the potential of a mosquito species to become an ecologically significant vector in the event of virus introduction, other factors affecting vectorial capacity need to be evaluated [62]: these factors include the presence of suitable hosts, mosquito longevity and biting rates and the impact of environmental temperatures. All three viruses have complex enzootic cycles involving more than one vertebrate host and more than one mosquito vector. Importantly, epizootic outbreaks of VEEV and RRV may occur involving equines or humans as the major vertebrate hosts. Humans are considered potential transport hosts for RRV [22] and rarely, VEEV [63]. By contrast, humans and equines are considered to be dead-end host of JEV, not able to produce a high enough viraemia to infect mosquitoes. Pigs are considered amplification hosts for JEV and experimental pig-pig transmission has been observed [50]. Therefore, it is at least theoretically possible for a mammal-biting mosquito such as *Oc. detritus* to be a vector of all three viruses in an epizootic outbreak.

The risk of transmission of equine arboviruses in the UK, including consideration of the potential for virus introduction or emergence [1, 13, 64] as well as the ecology of British mosquito species which may be considered candidate vectors, is discussed elsewhere [65]. Here, we focus on the mosquito species used in this study in relation to the ecological attributes which make them of interest as candidate vectors for the viruses tested. *Ochlerotatus detritus* is considered the primary species associated with brackish water that causes biting nuisance for humans in the UK [66, 67] and was trapped on seven of nine saltmarsh associated equine premises in the UK in a recent study [68]. Natural exposure of horses to *Oc. detritus* on the Wirral Peninsula, UK, has been used for testing of mosquito repellents, confirming regular blood-feeding from horses [65]. *Culiseta annulata* can be a locally significant nuisance species, noted particularly in early spring and late autumn in the UK, breeds in a variety of natural and artificial habitats, both shaded and unshaded and is widespread [66, 69]. *Culiseta annulata* has been shown to bite horses and other large animals in the UK [65, 68, 70] and engorged females have also been captured in horse baited traps in France and Switzerland [61, 71]. This species was found on 75% (24/32) equine premises sampled in a previous study in the UK [68]. Biting nuisance was experienced on two such premises during sampling, which was strongly suspected to be related to poorly drained muck-heaps which contained high densities of larvae identified as *Cs.* *annulata/alaskaensis/subochrea* and this species is associated with manure and water with a high nitrogen content [69, 72]. *Culiseta annulata* takes blood meals from birds as well as mammals including swine (an amplification host for JEV), both in the UK and elsewhere [73, 74] and has been considered a potential bridge-vector for WNV [75, 76]. *Culex pipiens* are considered abundant and widespread in the UK [75] and were found on 65% (15/23) of equine premises where suitable water sources were found in a previous study [68]. On all but four of these sites, mammal biting (candidate bridge-vector) species such as *Cs. annulata* or *Oc. detritus* were also trapped. In addition, *Culex pipiens* and/or *torrentium* mosquitoes were identified in horse-baited traps in the UK during testing for efficacy of repellents in a rural location, although these were not blood-fed [65]. In the UK, *Culex pipiens* ecoforms *pipiens* and *molestus*, and hybrids are present. The complexity of taxonomy of *Cx. pipiens* is discussed elsewhere [77] and here we use the term *Cx. pipiens* as mosquitoes were differentiated from *Cx. torrentium* but no attempt was made to define which ecoform they represented. For JEV, bird-mosquito-bird and bird-mosquito-mammal (bridge vector) transmission are likely to be required for ongoing transmission in the event of virus emergence, therefore vector competence studies of UK populations of ornithophilic species such as *Cx. pipiens* would provide important information. Overall, due to their widespread distribution and relative abundance, *Cx. pipiens,* whether ornithophilic or more catholic in their feeding preferences, must be considered a candidate vector (enzootic or as a bridge-vector) for JEV and here we demonstrate its ability to produce viral RNA in saliva at lower temperatures than previously shown.

Considering the current UK climate, the risk of enzootic establishment of these viruses appears low, however if climate change substantially alters factors such as the distribution, density and vectorial capacity of potential mosquito vectors then the risk of epizootic transmission may increase. The lowest maintenance temperature used in this study was 18 °C at which temperature, *Cx. pipiens* was able to produce JEV RNA in saliva. Assessment of vector potential at lower temperatures would be required in order to inform risk assessment for current UK climate conditions, although it is important to note that since the year 2000 there have been 10 years where July or August, or both, have had a mean temperature > 18 °C in East Anglia and 8 years for south east and the central south of England [78]. The 2.2 km convection permitting model (part of UKCP18) using the high emission scenario (RCP8.5) suggests that UK summer temperatures will rise by 3.6–5 °C for 2061–2080 [79] therefore assessment, at 21 °C was considered relevant to predicted future climate conditions. We detected viral RNA in saliva in mosquitoes incubated at 21 °C for all three virus-mosquito combinations.

As discussed previously, investigation of mosquito species’ potential for laboratory transmission of arboviruses by detection of viral RNA in saliva must be treated with a degree of caution and evaluation of vector competence and (even more so) estimation of potential vectorial capacity require additional information. Limitations of the present study include the relatively low numbers of *Cx. pipiens* used, lack of survival data and limited range of temperatures and time-points.

Further work which would be useful in evaluating the ability of UK populations of these mosquito species to transmit RRV, JEV or VEEV would include (but is not limited to): confirmation of production of infectious virus in saliva using cell titration methods; investigation of lower temperatures and shorter incubation times than those used in this study; and investigation of the apparent instability of mosquito infections, including at lower temperatures than those used in this study, to assess infection dynamics at temperatures to which these populations are adapted.

## Conclusions

The present study demonstrated that mosquito populations present in the UK are able to produce viral RNA in saliva after feeding on blood containing arboviruses which affect people and equines, and which are associated with significant morbidity and / or mortality in both groups. For all mosquito-virus pairs viral RNA was produced in the saliva of some mosquitoes. *Ochlerotatus* *detritus* demonstrated the ability to produce RRV RNA in saliva and low numbers produced VEEV RNA in saliva. *Culiseta annulata* and *Cx.* *pipiens* produced JEV RNA in saliva. For some mosquito-virus pairs there was evidence that infections were unstable and viral RNA decreased over time. Further work on the lower temperature limit for replication of JEV in *Cx. pipiens*, and confirmation that the RNA in saliva is indicative of infectious virus is warranted.

## Supplementary information

**Additional file 1: Table S1.** Table of C_q_ values for all mosquito virus pairs.

**Additional file 2: Text S1.** Derivation of ‘estimated relative quantity’ of RNA from C_q_ values.

## Data Availability

The datasets supporting the conclusions of this article are included within the article and its additional files.

## Abbreviations

cDNA: complementary deoxyribonucleic acid
C_q_ value: quantification cycle
df: degrees of freedom
EEEV: Eastern equine encephalitis virus
JEV: Japanese encephalitis virus
MVEV: Murray Valley encephalitis virus
nM: nanometers
NCPV: National Collection of Pathogenic Viruses
PCR: polymerase chain reaction
pfu: plaque forming units
P: probability value
qPCR: quantitative polymerase chain reaction
RCP: representative concentration pathway
RFLP: restriction fragment length polymorphism
RNA: ribonucleic acid
RRV: Ross River virus
SMICLD_50_: fifty per cent suckling mouse intracerebral lethal dose
TCID_50_: 50% tissue culture infectious dose
UKCP: United Kingdom Climate Projections
VEEV: Venezuelan equine encephalitis virus
WNV: West Nile virus

## References

[1] Chapman GE, Baylis M, Archer D, Daly JM (2018). The challenges posed by equine arboviruses. Equine Vet J..

[2] Papa A (2017). Emerging arboviral human diseases in Southern Europe. J Med Virol..

[3] Sardelis MR, Dohm DJ, Pagac B, Richard GA, Turell MJ (2002). Experimental transmission of Eastern Equine Encephalitis Virus by *Ochlerotatus j japonicus* (Diptera: Culicidae). J Med Entomol..

[4] Sardelis MR, Turell MJ, Dohm DJ, O’Guinn ML (2001). Vector competence of selected North American *Culex* and *Coquillettidia* mosquitoes for West Nile virus. Emerg Infect Dis..

[5] Turell MJ, Dohm DJ, Sardelis MR, O’guinn ML, Andreadis TG, Blow JA (2005). An update on the potential of North American mosquitoes (Diptera: Culicidae) to transmit West Nile virus. J Med Entomol..

[6] Turell MJ, O’Guinn ML, Dohm DJ, Jones JW (2001). Vector competence of North American mosquitoes (Diptera: Culicidae) for West Nile virus. J Med Entomol..

[7] Attoui H, Mendez-lopez MR, Rao S, Hurtado-Alendes A, Lizaraso-Caparo F, Jaafar FM (2009). Peruvian horse sickness virus and Yunnan orbivirus, isolated from vertebrates and mosquitoes in Peru and Australia. Virol J..

[8] Tauro LB, Rivarola ME, Lucca E, Mariño B, Nunes MRT, Contigiani MS (2016). Bunyamwera virus, an emerging pathogen of veterinary importance in Argentina. J Equine Vet Sci..

[9] Tauro LB, Rivarola ME, Lucca E, Mariño B, Mazzini R, Cardoso JF (2015). First isolation of Bunyamwera virus (Bunyaviridae family) from horses with neurological disease and an abortion in Argentina. Vet J..

[10] van Niekerk S, Human S, Williams J, van Wilpe E, Pretorius M, Swanepoel R (2015). Sindbis and Middelburg Old World alphaviruses associated with neurologic disease in horses, South Africa. Emerg Infect Dis..

[11] de Vos CJ, Swanenburg M, Tafro N, van Roon A, Stenvers OFJ, Elbers ARW (2017). Animal health risk of legally imported exotic animals into the Netherlands in the period 2013–2014. Microb Risk Anal..

[12] de Vos CJ, Hoek CA, Nodelijk G (2012). Risk of introducing African horse sickness virus into the Netherlands by international equine movements. Prev Vet Med..

[13] Durand B, Lecollinet S, Beck C, Martínez-López B, Balenghien T, Chevalier V (2013). Identification of hotspots in the European Union for the introduction of four zoonotic arboviruses by live animal trade. PLoS ONE..

[14] Faverjon C. Risk based surveillance for vector-borne diseases in horses: combining multiple sources of evidence to improve decision making. PhD Thesis, Universite Blaise Pascal, Aubiere; 2015.

[15] Chapman GE, Archer D, Torr S, Solomon T, Baylis M (2016). Potential vectors of equine arboviruses in the UK. Vet Rec..

[16] Carrara A-S, Gonzales M, Ferro C, Tamayo M, Aronson J, Paessler S (2005). Venezuelan equine encephalitis virus infection of spiny rats. Emerg Infect Dis..

[17] Greene IP, Paessler S, Austgen L, Anishchenko M, Brault AC, Bowen RA (2005). Envelope glycoprotein mutations mediate equine amplification and virulence of epizootic Venezuelan equine encephalitis virus. J Virol..

[18] Zehmer RB, Dean PB, Sudia WD, Calisher CH, Sather GE, Parker RL (1974). Venezuelan equine encephalitis epidemic in Texas, 1971. Health Serv Rep..

[19] Russell RC (2002). Ross River virus: ecology and distribution. Annu Rev Entomol..

[20] Barton AJ, Bielefeldt-Ohmann H (2017). Clinical presentation, progression, and management of five cases of Ross River virus infection in performance horses located in Southeast Queensland: a longitudinal case series. J Equine Vet Sci..

[21] Lau C, Aubry M, Musso D, Teissier A, Paulous S, Desprès P (2017). New evidence for endemic circulation of Ross River virus in the Pacific Islands and the potential for emergence. Int J Infect Dis..

[22] Liu W, Kizu JR, Le Grand LR, Moller CG, Carthew TL, Mitchell IR (2019). Localized outbreaks of epidemic polyarthritis among military personnel caused by different sublineages of Ross River virus, Northeastern Australia, 2016–2017. Emerg Infect Dis..

[23] Stephenson EB, Peel AJ, Reid SA, Jansen CC, McCallum H (2018). The non-human reservoirs of Ross River virus: a systematic review of the evidence. Parasit Vectors..

[24] Impoinvil DE, Baylis M, Solomon T, Mackenzie JS, Jeggo M, Daszak P, Richt JA (2013). Japanese encephalitis: on the one health agenda. One health: the human-animal-environment interfaces in emerging infectious diseases.

[25] Kim H, Cha G-W, Jeong YE, Lee W-G, Chang KS, Roh JY (2015). Detection of Japanese encephalitis virus Genotype V in *Culex orientalis* and *Culex pipiens* (Diptera: Culicidae) in Korea. PLOS ONE..

[26] Su C-L, Yang C-F, Teng H-J, Lu L-C, Lin C, Tsai K-H (2014). Molecular epidemiology of Japanese encephalitis virus in mosquitoes in Taiwan during 2005–2012. PLoS Negl Trop Dis..

[27] Ravanini P, Huhtamo E, Ilaria V, Crobu MG, Nicosia AM, Servino L (2012). Japanese encephalitis virus RNA detected in *Culex pipiens* mosquitoes in Italy. Euro Surveill..

[28] de Wispelaere M, Desprès P, Choumet V (2017). European *Aedes albopictus* and *Culex pipiens* are competent vectors for Japanese encephalitis virus. PLoS Negl Trop Dis..

[29] ECDPC. *Aedes albopictus*—current known distribution: January 2019 European Centre for Disease Prevention and Control; 2019. https://www.ecdc.europa.eu/en/publications-data/aedes-albopictus-current-known-distribution-january-2019. Accessed 28 Dec 2019.

[30] Vogels CB, Göertz GP, Pijlman GP, Koenraadt CJ (2017). Vector competence of European mosquitoes for West Nile virus. Emerg Microbes Infect..

[31] Schulz C, Becker SC (2018). Mosquitoes as arbovirus vectors: from species identification to vector competence. Mosq Borne Dis..

[32] Blagrove MSC, Sherlock K, Chapman GE, Impoinvil DE, McCall PJ, Medlock JM (2016). Evaluation of the vector competence of a native UK mosquito *Ochlerotatus detritus* (*Aedes detritus)* for dengue, chikungunya and West Nile viruses. Parasit Vectors..

[33] Huber K, Jansen S, Leggewie M, Badusche M, Schmidt-Chanasit J, Becker N (2014). *Aedes japonicus japonicus* (Diptera: Culicidae) from Germany have vector competence for Japan encephalitis virus but are refractory to infection with West Nile virus. Parasitol Res..

[34] MacKenzie-Impoinvil L, Impoinvil DE, Galbraith SE, Dillon RJ, Ranson H, Johnson N (2014). Evaluation of a temperate climate mosquito, *Ochlerotatus detritus* (*Aedes detritus*), as a potential vector of Japanese encephalitis virus. Med Vet Entomol..

[35] Fernandez Z, Moncayo AC, Carrara AS, Forattini OP, Weaver SC (2003). Vector competence of rural and urban strains of Aedes (Stegomyia) albopictus (Diptera : Culicidae) from Sao Paulo State, Brazil for IC, ID, and IF subtypes of Venezuelan equine encephalitis virus. J Med Entomol..

[36] Smith DR, Carrara A-S, Aguilar PV, Weaver SC (2005). Evaluation of methods to assess transmission potential of Venezuelan equine encephalitis virus by mosquitoes and estimation of mosquito saliva titers. Am J Trop Med Hyg..

[37] Hesson JC, Lundström JO, Halvarsson P, Erixon P, Collado A (2010). A sensitive and reliable restriction enzyme assay to distinguish between the mosquitoes *Culex torrentium* and *Culex pipiens*. Med Vet Entomol..

[38] Solomon T, Ni H, Beasley DWC, Ekkelenkamp M, Cardosa MJ, Barrett ADT (2003). Origin and evolution of Japanese encephalitis virus in Southeast Asia. J Virol..

[39] R Core Team. R: A Language and Environment for Statistical Computing. Vienna: R Foundation for Statistical Computing; 2017. https://www.R-project.org/.

[40] Holm S (1979). A simple sequentially rejective multiple test procedure. Scand J Stat..

[41] Andreadis TG, Anderson JF, Vossbrinck CR, Main AJ (2004). Epidemiology of West Nile virus in Connecticut: a five-year analysis of mosquito data 1999–2003. Vector Borne Zoonotic Dis..

[42] Farajollahi A, Fonseca DM, Kramer LD, Kilpatrick AM (2011). ″Bird biting″ mosquitoes and human disease: a review of the role of *Culex pipiens* complex mosquitoes in epidemiology. Infect Genet Evol..

[43] Turell MJ, Morrill JC, Rossi CA, Gad AM, Cope SE, Clements TL (2002). Isolation of West Nile and Sindbis viruses from mosquitoes collected in the Nile Valley of Egypt during an outbreak of Rift Valley fever. J Med Entomol..

[44] Danielova A (1972). The vector efficiency of *Culiseta annulata* mosquito in relation to Tahnya virus. Folia Parasitol (Praha)..

[45] Rosen L, Gubler DJ, Bennett PH (1981). Epidemic polyarthritis (Ross River) virus infection in the Cook Islands. Am J Trop Med Hyg..

[46] Kay BH, Pollitt CC, Fanning ID, Hall RA (1987). The experimental infection of horses with Murray Valley encephalitis and Ross River viruses. Aust Vet J..

[47] Sucre H, Alvarez O, Justines G (1980). Transplacental transmission of Venezuelan equine encephalitis virus in horses. Am J Trop Med Hyg..

[48] Henderson BE, Chappell WA, Johnston JG, Sudia WD. Experimental infection of horses with three strains of Venezuelan equine encephalomyelitis virus. I. Clinical and virological Studies. Am J Epidemiol. 1971;93:194–205. 10.1093/oxfordjournals.aje.a121246 4397564

[49] Wang E, Bowen RA, Medina G, Powers AM, Kang W, Chandler LM (2001). Virulence and viremia characteristics of 1992 epizootic subtype IC Venezuelan equine encephalitis viruses and closely related enzootic subtype ID strains. Am J Trop Med Hyg..

[50] Ricklin ME, García-Nicolás O, Brechbühl D, Python S, Zumkehr B, Nougairede A (2016). Vector-free transmission and persistence of Japanese encephalitis virus in pigs. Nat Commun..

[51] Gresser I, Hardy JL, Hu SMK, Scherer WF (1958). Factors influencing transmission of Japanese B encephalitis virus by a colonized strain of *Culex tritaeniorhynchus* Giles, from infected pigs and chicks to susceptible pigs and birds. Am J Trop Med Hyg..

[52] Scherer WF, Moyer JT, Izumi T (1959). Immunologic studies of Japanese encephalitis virus in Japan: V. Maternal antibodies, antibody responses and viremia following infection of swine. J Immunol..

[53] Boyle DB, Dickerman RW, Marshall ID (1983). Primary viraemia responses of herons to experimental infection with Murray Valley encephalitis, Kunjin and Japanese encephalitis viruses. Aust J Exp Biol Med Sci..

[54] Nemeth N, Bosco-Lauth A, Oesterle P, Kohler D, Bowen R (2012). North American birds as potential amplifying hosts of Japanese encephalitis virus. Am J Trop Med Hyg..

[55] Mackenzie-Impoinvil L, Impoinvil DE, Galbraith SE, Dillon RJ, Ranson H, Johnson N (2015). Evaluation of a temperate climate mosquito, *Ochlerotatus detritus* (=*Aedes detritus*), as a potential vector of Japanese encephalitis virus. Med Vet Entomol..

[56] Aitken THG (1977). An *in vitro* feeding technique for artificially demonstrating virus transmission by mosquitoes. Mosq News..

[57] Lumley S, Hernández-Triana LM, Horton DL, Fernández de Marco MDM, Medlock JM, Hewson R, et al. Competence of mosquitoes native to the United Kingdom to support replication and transmission of Rift Valley fever virus. Parasit Vectors. 2018;11:308. 10.1186/s13071-018-2884-7 PMC5960175 29776384

[58] Colton L, Biggerstaff BJ, Johnson A, Nasci RS (2005). Quantification of West Nile Virus in vector mosquito saliva. J Am Mosq Control Assoc..

[59] Anderson SL, Richards SL, Smartt CT (2010). A simple method for determining arbovirus transmission in mosquitoes. J Am Mosq Control Assoc..

[60] Beasley D, Klingler K, Higgs S, Vanlandingham DL, Huang J, Fair J (2004). Real-time reverse transcriptase-polymerase chain reaction quantification of West Nile virus transmitted by *Culex pipiens quinquefasciatus*. Am J Trop Med Hyg..

[61] Balenghien T, Fouque F, Sabatier P, Bicout DJ (2006). Horse-, bird-, and human-seeking behavior and seasonal abundance of mosquitoes in a West Nile virus focus of southern France. J Med Entomol..

[62] Kramer LD, Ebel GD (2003). Dynamics of flavivirus infection in mosquitoes. Adv Virus Res..

[63] Morrison AC, Forshey BM, Notyce D, Astete H, Lopez V, Rocha C (2008). Venezuelan equine encephalitis virus in Iquitos, Peru: urban transmission of a sylvatic strain. PLoS Negl Trop Dis..

[64] Pearce JC, Learoyd TP, Langendorf BJ, Logan JG (2018). Japanese encephalitis: the vectors, ecology and potential for expansion. J Travel Med..

[65] Chapman GE. Mosquito-borne arboviruses of horses: vector presence, competence and disease prevention in the UK, PhD Thesis, University of Liverpool, Liverpool, UK; 2017.

[66] Medlock JM, Hansford KM, Anderson M, Mayho R, Snow KR (2012). Mosquito nuisance and control in the UK. Eur Mosq Bull..

[67] Clarkson MJ, Setzkorn C (2011). The domestic mosquitoes of the Neston area of Cheshire, UK. Eur Mosq Bull..

[68] Chapman GE, Archer D, Torr S, Solomon T, Baylis M (2017). Potential vectors of equine arboviruses in the UK. Vet Rec..

[69] Becker N, Petrić D, Zgomba M, Boase C, Madon M, Dahl C, Becker N, Petric D, Zgomba M, Boase C, Madon M, Dahl C (2010). Subfamily Culicinae. Mosquitoes and their control.

[70] Hernández-Triana LM, Brugman VA, Prosser SWJ, Weland C, Nikolova N, Thorne L (2017). Molecular approaches for blood meal analysis and species identification of mosquitoes (Insecta: Diptera: Culicidae) in rural locations in southern England, United Kingdom. Zootaxa..

[71] Schönenberger AC, Wagner S, Tuten HC, Schaffner F, Torgerson P, Furrer S (2016). Host preferences in host-seeking and blood-fed mosquitoes in Switzerland. Med Vet Entomol..

[72] Chapman JM, Ormerod WE (1966). The survival of *Toxoplasma* in infected mosquitoes. J Hyg (Lond)..

[73] Service MW (1971). Feeding behaviour and host preferences of British mosquitoes. Bull Entomol Res..

[74] Dezső DJ (1990). Host feeding pattern of some mosquitoes (Diptera: Culicidae) collected in animal houses in Hungary. Parasitol Hung..

[75] Medlock JM, Snow KR, Leach S (2005). Potential transmission of West Nile virus in the British Isles: an ecological review of candidate mosquito bridge vectors. Med Vet Entomol..

[76] Brugman VA, Hernández-Triana LM, England ME, Medlock JM, Mertens PPC, Logan JG (2017). Blood-feeding patterns of native mosquitoes and insights into their potential role as pathogen vectors in the Thames estuary region of the United Kingdom. Parasit Vectors..

[77] Brugman VA, Hernández-Triana LM, Medlock JM, Fooks AR, Carpenter S, Johnson N. The role of *Culex pipiens* L. (Diptera: Culicidae) in virus transmission in Europe. Int J Environ Res Public Health. 2018;15:389. 10.3390/ijerph15020389 PMC5858458 29473903

[78] Met Office. UK and regional series. Met Off. 2019. https://www.metoffice.gov.uk/research/climate/maps-and-data/uk-and-regional-series. Accessed 13 Jun 2020.

[79] Met Office. ukcp local 2.2km non-technical summary Met Office. 2019. https://www.metoffice.gov.uk/binaries/content/assets/metofficegovuk/pdf/research/ukcp/ukcp-local-2.2km-non-technical-summary.pdf. Accessed 13 Jun 2020.

[80] Vina-Rodriguez A, Eiden M, Keller M, Hinrichs W, Groschup MH (2016). A quantitative real-time RT-PCR assay for the detection of Venezuelan equine encephalitis virus utilizing a universal alphavirus control RNA. BioMed Res Int..

[81] Pyke AT, Smith IL, van den Hurk AF, Northill JA, Chuan TF, Westacott AJ (2004). Detection of Australasian flavivirus encephalitic viruses using rapid fluorogenic TaqMan RT-PCR assays. J Virol Methods..

[82] Ye J, Coulouris G, Zaretskaya I, Cutcutache I, Rozen S, Madden TL (2012). Primer-BLAST: a tool to design target-specific primers for polymerase chain reaction. BMC Bioinformatics..

