# Supplementary material for: Laboratory transmission potential of British mosquitoes for equine arboviruses
Source: Parasit Vectors. 2020 Aug 12;13:413. doi: 10.1186/s13071-020-04285-x (PMC7425075; doi:10.1186/s13071-020-04285-x)
Supplement: Supplementary file 2 — Additional file 2: Text S1. Derivation of ‘estimated relative quantity’ of RNA from Cq values. [file 13071_2020_4285_MOESM2_ESM.docx]

# **Derivation of ‘estimated relative quantity’ of RNA from C_q_ values.**

To aid the interpretation of C_q_ values on plots, C_q_ values are converted to ‘estimated relative RNA quantity’ compared to a sample with a C_q_ value of 40 for each viral RNA. This is then presented on a log_10_ scale where a C_q_ value of 40 was represented by a log value of approximately 1.

Although this method of presentation produces a scale similar to that used for viral titres, it is important to remember that this is not what is being reported: PCR results could be described as semi-quantitative as the method does not fulfil requirements for absolute quantification of viral RNA. Absolute quantification of viral RNA was beyond the scope of this study, and requires rigorous quality control of RNA samples, quantification calibrators such as synthetic RNA or other recognized standards and ideally, internal controls to monitor for reaction inhibitors.

A standard curve for the PCR was generated using 3 replicates of 10-fold serial dilutions with a dynamic range of 7 logs using the stock virus. The average C_q_ values produced from this qPCR run were plotted against corresponding virus titre to produce an equation and proportion of variance explained (R^2^) for the best fit line using Least Squares estimation:

Standard curve plot for RRV, including the equation for best-fit line and R-squared value.

Where:

$y=mx+b$

For RRV, then:

slope (m) = -3.4467

intercept (b) = 44.284

Efficiency of the PCR reaction is calculated thus:

$Efficiency= -1+{10}^{(- \frac{1}{slope} )}$

For RRV, Efficiency = 95.04%

For quantitative PCR the formula used to calculate copy number, is:

X_0_ = E_AMP_ ^(b - Cq)^

Where:

The standard curve equation (1) gives b and m

X_0_ = copy number

Here, ‘estimated relative RNA quantity’ has been used instead of RNA copy number:

Estimated relative RNA quantity = E_AMP_ ^(b – Cq)^

Where:

E_AMP_ = exponential amplification value (Table 3.2) = 10^(-1/m)^

C_q_ = arithmetic mean of technical replicate C_q_ values for each sample.

Therefore, for RRV:

E_AMP_ = ${10}^{(-\frac{1}{-3.4467})}$ = 1.95

And:

Estimated relative RNA quantity (X_0_) = log_10_ 1.95 ^(44.284 - Cq)^

So for RRV, a C_q_ value of 40 corresponds to 1.24 on the log10 scale, and a C_q_ value of 20 corresponds to a value of 7.04.

It can therefore be seen that this method of presentation is based on the same scale as might be used to present viral RNA copy number, but results presented in this study are an estimate of RNA quantity relative to the other samples.

Parameters of the TaqMan assays.

| Virus | Efficiency | Standard Curve Equation | R^2^ | Range of C_q_ values produced in standard curve | Amplification factor |
| --- | --- | --- | --- | --- | --- |
| JEV | 103.19% | y = -3.2477x + 43.108 | 0.9972 | 19.88-39.35 | 2.03 |
| RRV | 95.04% | y = -3.4467x + 44.284 | 0.9949 | 17.77-37.35 | 1.95 |
| VEEV | 91.66% | y = -3.5393x + 45.531 | 0.9978 | 17.80-38.61 | 1.92 |
